# Social Stratification, Diet Diversity and Malnutrition among Preschoolers: A Survey of Addis Ababa, Ethiopia

**DOI:** 10.3390/nu12030712

**Published:** 2020-03-07

**Authors:** Hanna Y. Berhane, Magnus Jirström, Semira Abdelmenan, Yemane Berhane, Beatrix Alsanius, Jill Trenholm, Eva-Charlotte Ekström

**Affiliations:** 1 Department of Women’s and Children Health, International Maternal and Child Health, Uppsala University, 751 85 Uppsala, Sweden; semiraaaciph@gmail.com (S.A.); yemaneberhane@gmail.com (Y.B.); jill.trenholm@kbh.uu.se (J.T.); Lotta.Ekstrom@kbh.uu.se (E.-C.E.); 2 Addis Continental Institute of Public Health, 26751/1000 Addis Ababa, Ethiopia; 3 Department of Human Geography, Lund University, 223 62 Lund, Sweden; magnus.jirstrom@keg.lu.se; 4 Department of Biosystems and Technology, Swedish University of Agricultural Sciences, 230 53 Alnarp, Sweden; Beatrix.Alsanius@slu.se

**Keywords:** malnutrition, diet diversity, pre-school children, social stratification, urban, Ethiopia

## Abstract

In Sub-Saharan Africa, being overweight in childhood is rapidly rising while stunting is still remaining at unacceptable levels. A key contributor to this double burden of malnutrition is dietary changes associated with nutrition transition. Although the importance of socio-economic drivers is known, there is limited knowledge about their stratification and relative importance to diet and to different forms of malnutrition. The aim of this study was to assess diet diversity and malnutrition in preschoolers and evaluate the relative importance of socioeconomic resources. Households with children under five (5467) were enrolled using a multi-stage sampling procedure. Standardized tools and procedures were used to collect data on diet, anthropometry and socio-economic factors. Multivariable analysis with cluster adjustment was performed. The prevalence of stunting was 19.6% (18.5–20.6), wasting 3.2% (2.8–3.7), and overweight/obesity 11.4% (10.6–12.2). Stunting, overweight, wasting and limited diet diversity was present in all social strata. Low maternal education was associated with an increased risk of stunting (Adjusted odds ratio (AOR): 1.8; 1.4–2.2), limited diet diversity (AOR: 0.33; 0.26–0.42) and reduced odds of being overweight (AOR: 0.61; 0.44–0.84). Preschoolers in Addis Ababa have limited quality diets and suffer from both under- and over-nutrition. Maternal education was an important explanatory factor for stunting and being overweight. Interventions that promote diet quality for the undernourished whilst also addressing the burgeoning problem of being overweight are needed.

## 1. Introduction

Childhood malnutrition remains a major public health problem in low-income settings. Currently, the prevalence of being overweight and obesity is increasing whilst a high prevalence of stunting is ever-present [1]. The co-existence of under- and over-nutrition is commonly referred to as the “double burden” of malnutrition [2]. In low- and middle-income countries, this double burden is precipitated by the ongoing occurrence of economic and demographic changes most evident in urban areas [3,4]. Rapid urbanization has contributed to a nutritional transition which is characterized by a shift in dietary patterns from high-carbohydrate, low-fat diets to the consumption of energy-dense, high-fat diets accompanied by decreasing levels of physical activity [3,5,6,7].

The presence of both under- and over-nutrition within the same community suggests an inequitable distribution of wealth and social resources [8]. Socioeconomic factors, mainly education and income, have been identified as common drivers of the different forms of malnutrition. Understanding the common drivers presents an opportunity for coming up with interventions that can simultaneously address all forms of malnutrition [9]. In low-income countries, children in the poorest households have been more affected by under-nutrition as they are exposed to suboptimal living conditions like poor sanitation while their better-off counterparts may have better living conditions but suffer from being overweight/obesity [8,10,11,12,13]. However, in many settings, there is evidence that the burden of obesity is shifting towards the more vulnerable populations as inexpensive high-energy-dense and ultra-processed foods are becoming more available, affordable, convenient and therefore more readily consumed [1,14,15,16,17]. These transitions are particularly pronounced in urban settings.

Higher maternal education has been associated with improved child feeding practices [18], and reduced risk for stunting, wasting and being underweight [19]. On the other hand, higher maternal education levels have also been associated with frequent consumption of highly processed and sugar-sweetened beverages [20]. Educated urban mothers who work outside the house have time constraints to integrate the household chores. Consequently, they are likely to revert to convenient and processed foods which can lead to their children being overweight/obese [21,22]. Another important factor that has been associated with diet diversity and nutritional status is household food security. Food insecure households have been associated with limited diet diversity and poor nutritional outcomes [23,24]. Understanding to what extent the underlying socioeconomic factors influence child diet and nutrition in the context of nutrition transitions is essential in informing contextually cognizant public health policies and intervention strategies.

In Ethiopia, progress has been made in improving child nutrition. For example, stunting has decreased considerably from 58% in 2000 to 38% in 2016 [25]. However, this improvement has not been achieved equally by all population groups. There have been regional variations [25] as well as differences in wealth strata [26,27], especially in urban areas, where socioeconomic disparities have led to a continually widening gap between rich and poor [28], as reflected in their diet and nutritional status. Therefore, understanding the manner in which socioeconomic disparities in rapidly urbanizing Addis Ababa affect children’s diets and nutritional outcomes will be highly relevant. Hence, the aim of this study is to describe urban preschoolers’ diet and nutritional status with a focus on the stratification and relative importance of socio-economic factors such as household wealth, food security and maternal education.

## 2. Methods

### 2.1. Study Design and Setting

This study is a cross-sectional population-based survey covering the entire city of Addis Ababa, Ethiopia. Ethiopia is the second-most populous country in Africa, and the fastest-growing economy in the region [29]. Currently, the urban population constitutes 16% of the country’s population and is expected to double by 2037 [30].

Addis Ababa, the capital city of Ethiopia, has an estimated 3.4 million inhabitants [31] and is home to one-fourth of the countries urban population [32]. Despite the city being the main hub for economic activity, contributing approximately 50% towards the national GDP, it faces many challenges: high rates of unemployment (23.5%), poor housing conditions, and severe inequalities among the socio-economic strata [28,32]. The city is administratively divided into 10 sub-cities and each sub-city has 10–15 woredas (districts).

### 2.2. Sampling

This study covered all 116 woredas using a multi-stage sampling strategy; first, each woreda was divided spatially into five equal sections to serve as a cluster, of which one was selected using a computer-generated simple random sampling. In each cluster, guided by an interval of every third household, the team visited 60 households. All households that had at least one child under the age of five years and a caregiver/mother who consented to participate were included in the study. Mothers who were not available after three consecutive visits were deemed ineligible. Anthropometric measurements were taken from all children under five in the selected household. For the dietary assessment, one child from each household was selected. If the household had more than one eligible child, one was randomly selected to serve as a reference (index); this was enabled through the Open Data Kit (ODK) software on tablets.

### 2.3. Data Collection

Data collection for this study was based on two rounds of population-based surveys. The first round of collection took place during the wet season, reflecting a lean period, and the second round took place during the dry season, reflecting the post-harvest period.

Data were collected using a structured pre-coded interviewer-administered questionnaire uploaded onto tablets. The items included in the questionnaire were socioeconomic factors such as demographics, education, household assets, food security, and food consumption. The questionnaire was first prepared in English and then translated into the Amharic language, the official language of Ethiopia. A bilingual expert panel composed of English and Amharic speakers was convened to translate the study tool [33].

Ten teams of field workers, each consisting of five data collectors and one supervisor, collected the data. Everyone in the team received two weeks of training on interviewing techniques, the questionnaire contents, anthropometric measurements, and the use of tablets for data collection. Field personnel in charge of anthropometric measurements were given training which included a theoretical explanation, demonstration of said skills, and practice sessions both in class and in a mock field setup. Standardization was done according to recommendations [34]. The entire field procedure was pretested in clusters outside of the study sample. Necessary modifications were done following the pretest, which mainly involved replacing ambiguous words. The field supervisors and the researchers were closely involved at every stage of the fieldwork. Data were sent directly to a password-protected server. Stata version 14 software was used for data cleaning, which involved applying logic checks and running frequencies [35].

### 2.4. Measurement

Anthropometric measurements were taken for each child. Weight and length/height of each child were measured according to the World Health Organization (WHO) standards [36]. The weight of each child, minimally clad and/or removing wet diapers, was measured to the nearest 0.1 kg using the United Nations Children’s Fund (UNICEF) electronic scale. Recumbent length or height was measured to the nearest 0.1 cm using the UNICEF model wooden board as per the WHO protocol.

The participant’s socio-demographic characteristics were summarized by sex (male or female), age of mother in years, age of the child in months, family size (2–4, 5–7, 8+), current marital status (married/living together, divorced/widowed/separated), sex of the household head (female/male), and whether the mother was involved in an income-earning activity (yes/no).

Socioeconomic resources for the purpose of this study were defined as maternal education, household wealth, and household food security. They were measured as follows:

Maternal education was assessed by asking what the highest level of schooling completed by the mother at the time of the survey was. The level of education was then grouped into five categories: never attended/not finished first grade, grade 1–4, grade 5–8, grade 9–12, and college-educated, reflecting the Ethiopian educational system [37].

The household wealth index was constructed using principal component analysis (PCA). The indicator variables included were: ownership of house, type of housing unit, housing material (floor, roof, wall material), access to separate toilet facility and clean drinking water, as well as assets such as a bicycle, motorbike, car, cell phone, radio, TV, refrigerator, bed, Metad (electric stove used for making a local bread called Injera) and a savings account. Principal components with eigenvalues greater than one were retained to construct wealth index values and then categorized into wealth tertiles (low, medium and high) to serve as relative measures of household economic status [38].

The household food security status was assessed using the Household Food Insecurity Access Scale (HFIAS). A 1-month recall period was used to assess the food security of households. The household was categorized as food secure if it had not experienced any food insecurity conditions or had rarely worried about not having enough food, whereas food-insecure households were categorized as mild, moderate and severe in accordance with the guidelines [39].

### 2.5. Outcome Variables

The dietary assessment followed the Food and Agriculture Organization (FAO) recommendations [40]. First, the mothers/caretakers were asked to provide a 24-h recall of foods consumed by the child both at home and outside the home. For each item, the mother was asked whether the child consumed more than a spoonful. Once the mother completed listing the foods, including all the ingredients, she was shown pictures of common foods from each food group to help her recall and verify the food her child consumed within the past 24 h. The child food groups were developed based on the food items recommended in the Infant and Young Child Feeding (IYCF) guidelines. Total dietary diversity score (which was a count of “yes” response for the 7 food groups the child consumed) was calculated for each child. In accordance with the IYCF guidelines, children were considered to have adequately diversified dietary intake if they had at least four of the seven food groups, and those who had 3 or less of the food groups were considered to have inadequate diversity [41].

Anthropometric indices were calculated using the WHO Anthro software [42]. The Z-scores of indices height-for-age Z-score (HAZ), and weight-for-height Z-score (WHZ) were categorized using the WHO child growth standards. A child with a HAZ less than −2 standard deviations (SD) was defined as stunted, while those with WHZ less than −2 SD from the reference population were classified as wasted and +2 SD as overweight/obese [36].

### 2.6. Statistical Analysis

Data analyses were done using Stata version 14 [35]. Frequencies and percentages were calculated for all categorical variables. Cross-tabulation with a chi-square test for association and linear trend was done. Further, three statistical models were tested to evaluate independent effect associations while adjusting for potential confounders. The first model assessed the association of child nutritional status and diet diversity with each of the selected socioeconomic variables (household wealth, maternal education, household food security, and child sex) individually. The second model controlled for potential confounders (maternal age and child age) and the third model included both the potential confounders and all four socio-economic resources of interest. The generalized equation estimate (GEE) was used in all three models to estimate the crude odds ratios, and the adjusted odds ratios (AOR) along with their respective 95% confidence intervals (95% CI). All models were adjusted for clustering and the level of significance was set at *p*-value (<0.05). Multicollinearity was checked using the variance inflation factor (VIF) with the cut off set at below 5.

### 2.7. Ethical Considerations

The study protocol was approved by the institutional review board of Addis Continental Institute of Public Health Ref No. ACIPH/IRB/004/2015 on 15 December 2015. Permission was granted by all the sub-cities and woreda level health offices to facilitate the fieldwork. Each study participant was provided with comprehensive information about the objectives and goals of the research and oral consent was obtained prior to the data collection.

## 3. Results

**Figure 1:**
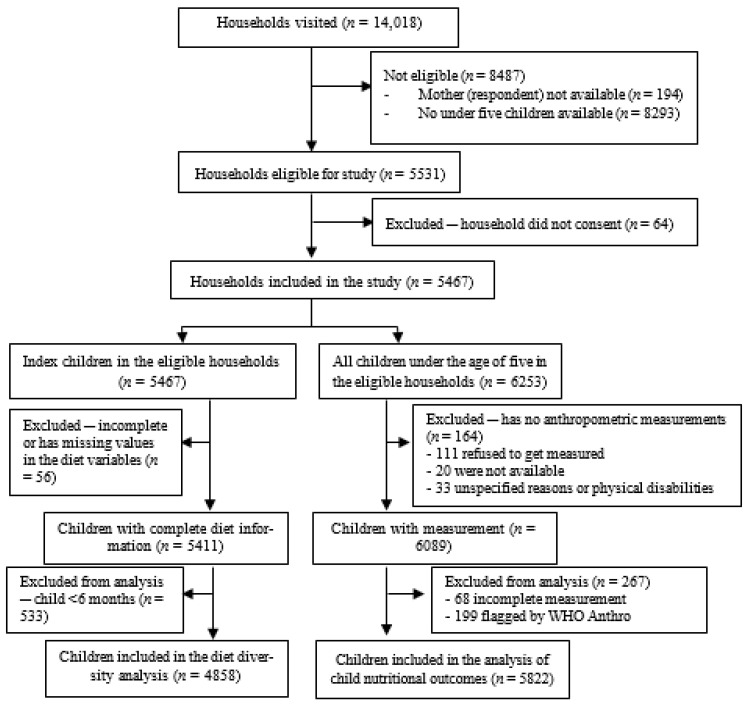
Flowchart of households and children included in the diet and child nutrition study in Addis Ababa, Ethiopia.

A total of 14,018 households were visited in two rounds (Figure 1). In 194 of those households, no eligible respondent (mother/caregiver) was available after three visits, and no eligible children (under the age of five years) were found in 8293 of the visited households. Amongst the households eligible for the study, 64 refused to participate. Hence, data were collected from 5467 eligible and available households. In those households, a total of 6253 children below the age of five years were identified. Complete anthropometric measurements were taken from 6089 of the 6253 children; 164 were not measured due to various reasons (111 refused, 20 were not available at the time of the study and 33 due to other reasons, such as physical disabilities). Furthermore, sixty-eight of the anthropometric measurements were omitted because they were incomplete and 199 (3.3%) excluded from the analysis because the anthropometric indicators (z-score) were flagged by the WHO Anthro software version 3.1. Child diet diversity scores were calculated from 4858/5467 children; this was done after excluding data that had one or more missing values in the food groups (*n* = 56) and/or had children below the age of six months (*n* = 553) (Figure 1).

The majority of households in this study were male-headed (86.5%), food secure (61.5%), and had a family size of 2–4 individuals (65.1%). More than half of the mothers were within the age group of 25–34 years (61.1%), 88% were married/living together and 77.2% had at least completed primary school (grade 5 and above). Among the children involved in this study, 52% were male and their mean age was 25.8 ± 15.5 months (Table 1).

### 3.1. Child Nutritional Status and Associations with Socioeconomic Resources

The prevalence of wasting was 3.2% (95% CI 2.8–3.7%), stunting was 19.6% (95% CI 18.5–20.6%), and being overweight/obesity was 11.4% (95% CI 10.6–12.2%) (Table 2). Both forms of malnutrition were present in all three socio-economic resources and across the respective strata. Furthermore, increasing and decreasing trends in the prevalence of stunting and being overweight were found within household wealth, maternal education and food security, but there was no social stratification for wasting (Table 2).

Maternal education showed the largest observed difference on social stratification. Children of mothers with an educational level of college or grade 9–12 had a lower prevalence of stunting (14.4 and 17.6%, respectively) than children born to mothers with lower educational levels (21.8–24.3%). On the other side of the malnutrition spectrum, a college-level education was also associated with a higher prevalence of being overweight (15.4%) than those children born to the mothers with lower levels of education (8.2–11.3%). The maternal educational level did not show an association with wasting (Table 2).

Food security displayed a similar social stratification as maternal education, where children living in food-secure households had a lower prevalence of stunting (17.5%) than children in moderate and severely food insecure households (21.7 and 26.7%, respectively). The food secure children also had a higher prevalence of obesity (12.7%) than children in moderate or severely food insecure households (9.2% and 8.7%, respectively). Food security did not show a social stratification for wasting (Table 2).

With regards to wealth, children from the highest wealth tertile had a significantly lower prevalence of stunting (17.3%) and a higher prevalence of obesity (13.2%) than children from the lowest wealth strata; this follows a similar pattern as the other two socioeconomic resources. Boys had both a higher prevalence of stunting and wasting compared to girls (Table 2).

After adjusting for food security and wealth, maternal education was inversely associated to both extremes of children’s malnutrition, where the odds of being stunted were almost two times higher (AOR: 1.8, 95% CI: 1.4–2.2) and the odds of being overweight/obese were lower (AOR: 0.61, 95% CI: 0.44–0.84) in children whose mothers never attended/finished first grade compared to those who were college-educated (Table 3). For food security, adjusted analyses revealed that stunting was associated with household food insecurity, where odds of stunting were 1.4 times higher among children from severely food insecure households (AOR: 1.42, 95% CI: 1.14–1.76). Strata of food security had no independent association with being overweight/obesity status (Table 3). Regarding wealth, children in the highest wealth households had higher odds of being overweight/obese, while no significant association was observed between household wealth and stunting. (Table 3).

Finally, with regard to the sex of the child, boys had a higher risk for stunting (AOR: 1.2, 95% CI: 1.1–1.4) and wasting (AOR: 1.38, 95% CI: 1.03–1.86) but no significant association was observed between the sex of the child and overweight/obesity status (Table 3).

### 3.2. Child Diet Diversity and Associations with Socioeconomic Resources

The proportion of children who had consumed at least 4 food groups (minimum recommended dietary diversity) was about 60% (59.9%; 95% CI: 58.5–61.3). Inadequate diet diversity appeared in all three socio-economic resources and showed differences across all strata. More children from the highest wealth households received an adequate diet diversity compared to children in the lowest (70% vs. 49%). The difference in the prevalence of adequate diet diversity was more than 30% between the food secure (68%) as compared to the severely food insecure (33%), and among children who had college-educated mothers (75%) as compared to those who had never attended school (42%). However, there was no difference by sex (Table 4).

When evaluating the independent associations, all three socio-economic resources showed significant variations in diet diversity (Table 4). Compared to children in the highest wealth tertile, children in the lowest wealth tertile had lower odds of having a diverse diet (AOR: 0.60, 95% CI: 0.51–0.71). Adequate diet diversity was attributed to maternal education and household food security. Children with mothers who never attended school and those from food-insecure households had lower odds of having adequate diet diversity. No significant association was observed between the diet diversity of the different sexes (boys vs. girls) (Table 4).

## 4. Discussion

The results of the study showed that 40% of the participating children had inadequate diet diversity and that the prevalence of being overweight/obesity was high, stunting was moderate, and wasting was low. Limited diet diversity and both forms of malnutrition were present in all socioeconomic resources, with the exception of wasting, where no significant variation was observed within all strata. The highest difference was observed when stratified by maternal education level, and was associated with diet diversity, stunting, and being overweight/obesity. After controlling for potential confounders, food security was significantly associated with diet diversity and stunting, while wealth was significantly associated with diet diversity and being overweight. Although there was no difference in boys’ and girls’ diet diversity, boys were more prone to both stunting and wasting than girls.

Our results show the presence of the dual burden of malnutrition: a high prevalence of being overweight/obesity co-existing with under-nutrition. The rates of wasting (3.2%) were low and close to meeting the target of maintaining wasting below 3% by 2030 set by the Sustainable Development Goals (SDG) [43,44]. However, stunting results were not as promising. Compared to the national estimates for Addis, this study reports a prevalence which is approximately 5% higher [25]. Being overweight/obesity was also high in the study population [44], in particular when compared with the prevalence reported in the Ethiopian Demographic and Health Survey (EDHS) 2016 for Addis Ababa (7%) [25]. The differences in our observed prevalence as compared to the EDHS could be due to the small sample size used in the EDHS for Addis (*n* ≤ 220), which may not reflect the child population of the city. However, our study’s results are comparable with a pooled prevalence from a meta-analysis done in 2018 which shows that Addis Ababa had the highest obesity rates among the regions with a pooled prevalence of 11.94 (95% CI: 9.39–14.50) [45].

Four out of ten children in this study were not receiving an adequately diverse diet. This finding is consistent with national figures indicating that children in Ethiopia have a monotonous diet, with more than 80% of children not receiving adequate diet diversity [25]. Supporting this, the food consumption survey also showed that cereals/roots and tubers constitute the major portion of the diet and carbohydrates contribute to 67.2%, of the total energy intake [46]. The relatively high dietary diversity we reported here could be due to the fact that in both the aforementioned national surveys, only younger age groups were included (6–35 months) than in this study context which included 6–59 month olds. Younger children are subjected to more dietary restrictions such as not being given meat and/or dairy products (except milk) due to the common belief that they are too young to chew and digest these items [47].

Although both forms of malnutrition were present in children with educated/uneducated mothers, children with an educated mother had lower odds of stunting and higher odds of being overweight/obesity. Similarly, children with educated mothers had better diet diversity. Previous findings have also shown that children with educated mothers had better dietary diversity [48] and lower odds of stunting [49,50,51,52]. Several mechanisms through which maternal education affects dietary diversity and nutrition have been identified. Educated mothers are more likely to better understand nutrition information, they have better antenatal care follow-up, they make use of family planning, and they generally exhibit more health-seeking behavior [25,53]. Having an educated mother was also associated with a higher likelihood of being fed a diverse diet [54]. Moreover, educated mothers were involved in income-earning activities, enabling them to purchase a diverse diet for their child. This could also influence autonomous decision-making ability within the household, which has been shown to improve dietary and nutrition outcomes for children [55,56]. Conversely, children with college-educated mothers were also found to have higher odds of being overweight/obesity. A plausible explanation for this could be that mothers who are educated were more likely to be working outside the home and may lack the time to cook, therefore opting for convenient pre-prepared processed packaged foods [21,22]. Another explanation could be that working mothers would have more financial liberty to buy more “treats”, as in highly processed and sugar-sweetened beverages [20], which could lead to children being overweight/obese.

Household food insecurity was associated with the child lacking diet diversity and stunting. The difference in diet diversity for children from food secure (68%) versus food insecure (33%) households was double. This difference was also significant after adjusting for possible confounders. Given that Household Food Insecurity Access Scale (HFIAS) is an indicator of the households’ experience of limitations in accessing food, this difference may not be surprising. Other studies have also shown that children from severely food insecure households rarely have an adequately diverse diet [23,24]. Stunting was also significantly higher among children from food insecure households. Consistent with this finding, severe food insecurity has been associated with severe stunting and wasting [57]. Persistent food insecurity has been linked with decreased intake, monotonous diet and stunting [23,57].

With regard to household wealth, children from the highest wealth strata had higher odds of getting an adequate diet, as well as being overweight. The prevalence of diet diversity was much higher—nearly a 20% difference between the lowest and highest wealth tertiles—which remained significant after adjustments. Another Ethiopian study also showed that improved dietary diversity is positively associated with household wealth [48] as food choices/diet are driven by price [58]. The increase in being overweight/obesity, despite having a better diet diversity, has been described by Hawkes [9] as the “double-edged sword of malnutrition” because being in a higher wealth strata is likely to increase the risk of being overweight/obesity even though the risk of stunting is reduced. A likely explanation could be that the well-off group can afford to purchase and consume high energy-dense foods and snacks that are made available by the changing and expanding market [59,60]. Another plausible explanation for the increase in obesity among children from wealthier households could be that they have more access to technological devices like televisions which encourage passivity instead of playing outdoors and spending time in more active pursuits [61].

This study showed that despite similarities in diet diversity, the odds of being wasted and stunted were higher among boys compared to girls. A similar association was observed in a meta-analysis of 16 demographic and health surveys [62], whereby malnutrition among boys is consistently higher than girls. One explanation could be biological—boys require more caloric intake compared to girls, making them more susceptible to stunting (especially in resource-limited households). Additionally, evidence has shown that at earlier ages, boys are more prone to infection than girls [63]. Furthermore, in another study, mothers’ perceptions that their male children were hungrier pushed them to initiate supplemental foods earlier, leading to a higher susceptibility to infections/diarrheal disease [64].

For many years, undernutrition has justly dominated the Ethiopian malnutrition discussions for many reasons. However, this research has revealed that Addis Ababa’s high prevalence of overweight preschoolers constitutes a public health issue that also requires attention. Hence, creating tailored interventions to curb the double burden of malnutrition is both challenging and critical. WHO has proposed so-called “double duty actions” which include promoting exclusive breastfeeding for the first six months, as well as promotion of appropriate complementary feeding, better maternal nutrition and antenatal care, school food programs, and specific marketing regulations [65]. Although Ethiopia has been focusing on reducing under-nutrition, still there are several national policies and initiatives that are in line with these WHO recommendations. These include the initiation of national school feeding programs for primary school students, regulations on formula milk quality and labeling, and free antenatal and delivery services at health centers. Despite these efforts, Ethiopia is lagging on some of the key indicators such as exclusive breastfeeding for the first six months (currently at 58%), antenatal care visits (four or more visits) is at 32%, and only 7% of children aged 6–23 months receive the minimum acceptable diet [25]. These statistics suggest the need for adapting new approaches and strategies.

Given that maternal education was the socioeconomic resource that showed the strongest association with measures of both under- and over-nutrition, as well as the delivery of a quality diet, focusing on maternal education could present one such opportunity. Ethiopia is one of the countries that provide free education, featuring a primary education coverage of 90%, although it drops drastically to 30% in secondary school [25]. There are country-specific thresholds signifying the number of years of schooling needed for a significant reduction in child malnutrition [66]; accordingly, Ethiopia also needs to develop a contextual threshold level because primary education alone may not be enough to make the needed nutritional impact. Behavioral interventions and health promotion initiatives tailored to the mother’s level of understanding could be one opportunity, however, there is limited evidence on its implementation and success in tackling under- and over-nutrition simultaneously [67].

This study has some limitations. First, weighting of prevalence was not done in relation to cluster size. Therefore, estimates may have been inflated/deflated, that is, if there was an over- or under-sampled segment of the population. However, only a modest effect on estimates was expected as Addis Ababa is assumed to have an equal distribution of population size by district. Second, although we used standard tools in combination with novel photo-based approaches to minimize recall and social desirability bias, there is the possibility that the child’s actual intake was affected by social desirability or underestimated by mothers who expected support and overestimated if she was aware of the perils of a monotonous diet. One of the strengths of this study is its large sample size and the inclusion of all administrative sub-units. The cities in Ethiopia have similar socioeconomic challenges [68] and in that sense, these findings may also be applicable to them. It may be plausible to cautiously relate these findings to other rapidly urbanizing cities in Sub-Saharan Africa depending on their contexts. Anthropometric measurements were performed under standardized measurement protocol and therefore would have high reliability.

## 5. Conclusions

The co-existence of both under- and over-nutrition was evident among preschoolers in Addis Ababa; being overweight has reached significantly high levels [44], constituting an important public health risk, while poor diet and stunting remain as pressing issues. Limited dietary diversity, child stunting, and being overweight were common features among the pre-school children of Addis Ababa regardless of wealth, maternal education and household food security. Higher maternal education appeared to protect against low diet diversity and stunting but was also associated with higher odds of being overweight. Interventions to improve pre-school children’s nutrition and food security needs to be very carefully tailored to navigate between the need to increase diet quality and reduce stunting while minimizing the risk of being overweight.

## Figures and Tables

**Table 1:** General households, maternal and child characteristics in Addis Ababa, Ethiopia.

Level	Characteristics (*n* = 5467)	*n*	%
Household	Male headed households	4729	86.5
	2 to 4 family size	3561	65.1
	Housing ownership (*n* = 5452)		
	Privately owned	1165	21.3
	Rented from private	2329	42.6
	Rented from public	1360	24.9
	Rent-free	598	11.0
	Household food insecurity		
	Food secure	3362	61.5
	Mildly food insecure	500	9.2
	Moderately food insecure	1070	19.6
	Severely food insecure	535	9.8
Maternal	Age		
	15–24	864	15.8
	25–34	3342	61.1
	35–44	999	18.3
	45+	262	4.8
	Marital status		
	Married/living together	4813	88.1
	Never married/divorced/widowed/separated	654	11.9
	Education		
	Never attended/ finished first grade	752	13.8
	Grade 1–4	498	9.1
	Grade 5–8	1638	30.0
	Grade 9–12	1482	27.1
	College	1097	20.1
	Involved in income-earning activity	1432	26.2
Child	Sex (Male)	2847	52.1
	Age		
	0–5 months	556	10.2
	6–11 months	657	12.0
	12–23 months	1337	25.0
	24–35 months	1286	23.5
	36–47 months	945	17.3
	48–59 months	686	12.6
	Nutritional status (*n* = 5822)		
	Stunted	1139	19.6
	Wasted	187	3.2
	Overweight/Obese	664	11.4
	Diet diversity (>3 food groups)	2911	59.9

Definitions: stunted (height-for-age Z-score < −2 standard deviations (SD)); wasted (weight-for height Z-score < −2 SD); and overweight/obese (weight-for height Z-score > +2 SD).

**Table 2:** Prevalence of child malnutrition by socio-economic resources in Addis Ababa, Ethiopia.

	Stunted	Overweight/Obese	Wasted
	*n* (%)	*n* (%)	*n* (%)
All	1139 (19.6)	664 (11.4)	187 (3.2)
Household wealth			
Lowest tertile (*n* = 1935)	428 (22.1)	187 (9.7)	62 (3.2)
Middle tertile (*n* = 1953)	377 (19.3)	222 (11.4)	58 (3.0)
Highest tertile (*n* = 1934)	334 (17.3)	255 (13.2)	67 (3.5)
	*p* = 0.0001	*p* = 0.001	*p* = 0.17
Mother’s Education			
Never attended/less than first grade (*n* = 782)	190 (24.3)	64 (8.2)	28 (3.6)
Grade 1–4 (*n* = 528)	120 (22.7)	57 (10.8)	10 (1.9)
Grade 5–8 (*n* = 1732)	377 (21.8)	180 (10.4)	62 (3.6)
Grade 9–12 (*n* = 1582)	279 (17.6)	178 (11.3)	45 (2.8)
College (*n* = 1198)	173 (14.4)	185 (15.4)	42 (3.5)
	*p* = 0.0001	*p* = 0.0001	*p* = 0.91
Household Food Insecurity			
Food secure (*n* = 3581)	625 (17.5)	454 (12.7)	105 (2.9)
Mildly insecure (*n* = 533)	115 (21.6)	56 (10.5)	22 (4.1)
Moderately insecure (*n* = 1135)	246 (21.7)	104 (9.2)	40 (3.5)
Severely insecure (*n* = 573)	153 (26.7)	50 (8.7)	20 (3.5)
	*p* = 0.0001	*p* = 0.0001	*p* = 0.26
Child Sex			
Male (*n* = 3018)	635 (21.0)	341 (11.3)	111 (3.7)
Female (*n* = 2804)	504 (18.0)	323 (11.5)	76 (2.7)
	*p* = 0.003	*p* = 0.069	*p* = 0.036

Abbreviations: CI, confidence interval.

**Table 3:** Association of socio-economic resources and child malnutrition in Addis Ababa, Ethiopia.

	Stunted			Overweight/Obese			Wasted		
	Unadjusted OR (95% CI) ^a^	Age-Adjusted OR (95% CI) ^b^	Fully-Adjusted OR (95% CI) ^c^	Unadjusted OR (95% CI) ^a^	Age-Adjusted OR (95% CI) ^b^	Fully-Adjusted OR (95% CI) ^c^	Unadjusted OR (95% CI) ^a^	Age-Adjusted OR (95% CI) ^b^	Fully-Adjusted OR (95% CI) ^c^
**Household Wealth**									
Lowest Tertile	1.37 * (1.16–1.60)	1.35 * (1.15–1.59)	1.14 (0.96–1.35)	0.71 * (0.58–0.86)	0.68 * (0.55–0.83)	0.77 * (0.62–0.96)	0.92 (0.65–1.31)	0.93 (0.65–1.33)	0.88 (0.61–1.29)
Middle Tertile	1.15 (0.98–1.36)	1.14 (0.97–1.35)	1.03 (0.87–1.23)	0.84 (0.69–1.02)	0.83 (0.68–1.01)	0.90 (0.74–1.10)	0.85 (0.59–1.22)	0.86 (0.60–1.23)	0.82 (0.57–1.19)
Highest Tertile	Ref	Ref	Ref	Ref	Ref	Ref	Ref	Ref	Ref
**Mother’s Education**									
Never Attended/ Finished First Grade	1.86 * (1.48–2.34)	2.00 * (1.57–2.54)	1.75 * (1.36–2.24)	0.49 * (0.36–0.67)	0.52 * (0.38–0.71)	0.61 * (0.44–0.84)	1.01 (0.62–1.65)	1.04 (0.62–1.72)	0.95 (0.55–1.62)
Grade 1–4	1.72 * (1.33–2.24)	1.73 * (1.33–2.26)	1.54 * (1.17–2.02)	0.67 * (0.49–0.92)	0.69 * (0.50–0.95)	0.78 (0.56–1.08)	0.53 (0.26–1.06)	0.54 (0.27–1.09)	0.50 (0.25–1.03)
Grade 5–8	1.61 * (1.32–1.97)	1.57 * (1.28–1.92)	1.46 * (1.19–1.80)	0.64 * (0.51–0.80)	0.65 * (0.52–0.81)	0.71 * (0.56–0.89)	1.01 (0.68–1.51)	1.04 (0.70–1.31)	1.01 (0.66–1.53)
Grade 9–12	1.25 * (1.02–1.54)	1.21 (0.98–1.48)	1.18 (0.96–1.45)	0.70 * (0.56–0.87)	0.74 * (0.59–0.93)	0.77 * (0.61–0.96)	0.80 (0.52–1.22)	0.85 (0.55–1.31)	0.84 (0.55–1.30)
College	Ref	Ref	Ref	Ref	Ref	Ref	Ref	Ref	Ref
**Household Food Insecurity**									
Severely Insecure	1.67 * (1.36–2.06)	1.69 * (1.37–2.08)	1.42 * (1.14–1.76)	0.67 * (0.49–0.91)	0.70 * (0.52–0.96)	0.84 (0.61-1.16)	1.20 (0.74-1.95)	1.22 (0.75-1.99)	1.30 (0.77-2.18)
Moderately Insecure	1.32 * (1.12–1.56)	1.32 * (1.12–1.56)	1.15 (0.97–1.38)	0.70 * (0.56–0.87)	0.70 * (0.56–0.88)	0.81 (0.63–1.02)	1.21 (0.84–1.76)	1.23 (0.84–1.78)	1.28 (0.87–1.91)
Mildly Insecure	1.30 * (1.04–1.63)	1.30 * (1.04–1.63)	1.22 (0.97–1.53)	0.81 (0.60–1.09)	0.81 (0.60–1.09)	0.87 (0.64–1.17	1.42 (0.89–2.28)	1.41 (0.88–2.27)	1.50 (0.93–2.42)
Food Secure	Ref	Ref	Ref	Ref	Ref	Ref	Ref	Ref	Ref
**Child Sex**									
Male	1.21 * (1.07–1.38)	1.21 * (1.06–1.38)	1.22 * (1.06–1.39)	0.98 (0.83–1.15)	0.99 (0.84–1.16)	0.98 (0.84–1.16)	1.37 * (1.02–1.84)	1.38 * (1.02–1.86)	1.38 * (1.03–1.86)
Female	Ref	Ref	Ref	Ref	Ref	Ref	Ref	Ref	Ref

Generalized estimating equations (GEE) with binomial family and exchangeable correlation structure; Abbreviations: OR, odds ratio; CI, confidence interval; Ref, reference group; * significance level of <0.05. ^a^ Model 1 = unadjusted; ^b^ Model 2 = Model 1 + adjusted for maternal and child age; ^c^ Model 3 = Model 2 + wealth tertile, household food insecurity, sex (child), maternal education; clustering effect was controlled for all models.

**Table 4:** Child diet and its association with socio-economic resources in Addis Ababa, Ethiopia.

		Minimum Dietary Diversity (≥4 Food Groups)		
	*n* (%)	Unadjusted OR (95% CI) ^a^	Age-Adjusted OR (95%CI) ^b^	Fully-Adjusted OR (95% CI) ^c^
All	2911 (59.9)			
**Household Wealth**				
Lowest Terile (*n* = 1616)	794 (49.1)	0.40 * (0.35–0.47)	0.40 * (0.34–0.46)	0.60 * (0.51–0.71)
Middle Tertile (*n* = 1647)	995 (60.4)	0.63 * (0.55–0.73)	0.62 * (0.53–0.72)	0.80 * (0.68–0.93)
Highest Tertile (*n* = 1595)	1122 (70.3)	Ref	Ref	Ref
**Mother’s Education**				
Never Attended/Less than First Grade (*n* = 688)	293 (42.6)	0.25 * (0.20–0.31)	0.21 * (0.17–0.27)	0.33 * (0.26–0.42)
Grade 1–4 (*n* = 440)	191 (43.4)	0.26 * (0.20–0.32)	0.24 * (0.18–0.30)	0.35 * (0.27–0.45)
Grade 5–8 (*n* = 1444)	811 (56.2)	0.43 * (0.36–0.51)	0.41 * (0.34–0.50)	0.52 * (0.43–0.64)
Grade 9–12 (*n* = 1338)	901 (67.3)	0.68 * (0.56–0.82)	0.66 * (0.54–0.80)	0.74 * (0.61–0.90)
College (*n* = 948)	715 (75.4)	Ref	Ref	Ref
**Household Food Security**				
Severely Insecure (*n* = 476)	158 (33.2)	0.24 * (0.20–0.30)	0.22 * (0.18–0.27)	0.32 * (0.26–0.40)
Moderately Insecure (*n* = 952)	466 (49.0)	0.44 * (0.38–0.51)	0.42 * (0.36–0.48)	0.56 * (0.47–0.66)
Mildly Insecure (*n* = 455)	251 (55.2)	0.58 * (0.47–0.70)	0.56 * (0.45–0.69)	0.68 * (0.55–0.84)
Secure (*n* = 2975)	2036 (68.4)	Ref	Ref	Ref
**Child Sex**				
Male (*n* = 2541)	1545 (60.8)	1.08 (0.97–1.21)	1.08 (0.96–1.21)	1.08 (0.96–1.23)
Female (*n* = 2317)	1366 (59.0)	Ref	Ref	Ref

Generalized estimating equations (GEE) with binomial family and exchangeable correlation structure; Abbreviations: OR, odds ratio; CI, confidence interval; Ref, reference group; * significance level of <0.05. ^a^ Unadjusted; ^b^ Adjusted for maternal and child age; ^c^ adjusted for maternal and child age, wealth tertile, sex (child), maternal education, food security; clustering effect was controlled for all models.

## References

[1] Tzioumis E., Adair L.S. (2014). Childhood dual burden of under- and over-nutrition in low- and middle-income countries: A critical review. Food Nutr. Bull..

[2] World Health Organization Double burden of malnutrition. https://www.who.int/nutrition/double-burden-malnutrition/en/.

[3] Crush J., Frayne B., McLachlan M. (2011). Rapid Urbanization and the Nutrition Transition in Southern African. Kingston and Cape Town: Queen’s University and AFSUN. https://fsnnetwork.org/sites/default/files/rapid_urbanization_and_the_nutrition.pdf.

[4] Popkin B.M., Corvalan C., Grummer-Strawn L.M. (2020). Dynamics of the double burden of malnutrition and the changing nutrition reality. Lancet.

[5] Popkin B.M. (2015). Nutrition Transition and the Global Diabetes Epidemic. Curr. Diabet. Rep..

[6] Popkin B.M. (2006). Global nutrition dynamics: The world is shifting rapidly toward a diet linked with noncommunicable diseases. Am. J. Clin. Nutr..

[7] Popkin B.M., Adair L.S., Ng S.W. (2012). NOW AND THEN: The Global Nutrition Transition: The Pandemic of Obesity in Developing Countries. Nutr. Rev..

[8] Perez-Escamilla R., Bermudez O., Buccini G.S., Kumanyika S., Lutter C.K., Monsivais P., Victora C. (2018). Nutrition disparities and the global burden of malnutrition. BMJ.

[9] Hawkes C., Ruel M.T., Salm L., Sinclair B., Branca F. (2020). Double-duty actions: Seizing programme and policy opportunities to address malnutrition in all its forms. Lancet.

[10] Kimani-Murage E.W., Muthuri S.K., Oti S.O., Mutua M.K., van de Vijver S., Kyobutungi C. (2015). Evidence of a Double Burden of Malnutrition in Urban Poor Settings in Nairobi, Kenya. PLoS ONE.

[11] Smith D.W. (1998). Urban Food Systems and the Poor in Developing Countries. Trans. Inst. Br. Geogr..

[12] Ziraba A.K., Fotso J.C., Ochako R. (2009). Overweight and obesity in urban Africa: A problem of the rich or the poor?. BMC Public Health.

[13] Van de Poel E., Hosseinpoor A.R., Jehu-Appiah C., Vega J., Speybroeck N. (2007). Malnutrition and the disproportional burden on the poor: The case of Ghana. Int. J. Equity Health.

[14] Rogers R., Eagle T.F., Sheetz A., Woodward A., Leibowitz R., Song M., Sylvester R., Corriveau N., Kline-Rogers E., Jiang Q. (2015). The Relationship between Childhood Obesity, Low Socioeconomic Status, and Race/Ethnicity: Lessons from Massachusetts. Child Obes..

[15] Drewnowski A., Moudon A.V., Jiao J., Aggarwal A., Charreire H., Chaix B. (2014). Food environment and socioeconomic status influence obesity rates in Seattle and in Paris. Int. J. Obes..

[16] Villanueva R., Albaladejo R., Astasio P., Ortega P., Santos J., Regidor E. (2016). Socio-economic environment, area facilities and obesity and physical inactivity among children. Eur. J. Public Health.

[17] Herforth A., Ahmed S. (2015). The food environment, its effects on dietary consumption, and potential for measurement within agriculture-nutrition interventions. Food Secur..

[18] Demilew Y.M., Tafere T.E., Abitew D.B. (2017). Infant and young child feeding practice among mothers with 0–24 months old children in Slum areas of Bahir Dar City, Ethiopia. Int. Breastfeed J..

[19] Akombi B.J., Agho K.E., Renzaho A.M., Hall J.J., Merom D.R. (2019). Trends in socioeconomic inequalities in child undernutrition: Evidence from Nigeria Demographic and Health Survey (2003–2013). PLoS ONE.

[20] Contreras M., Blandón E.Z., Persson L.-Å., Hjern A., Ekström E.-C. (2015). Socio-economic resources, young child feeding practices, consumption of highly processed snacks and sugar-sweetened beverages: A population-based survey in rural northwestern Nicaragua. BMC Public Health.

[21] Beshara M., Hutchinson A., Wilson C. (2010). Preparing meals under time stress. The experience of working mothers. Appetite.

[22] Jabs J., Devine C.M., Bisogni C.A., Farrell T.J., Jastran M., Wethington E. (2007). Trying to Find the Quickest Way: Employed Mothers’ Constructions of Time for Food. J Nutr. Educ. Behav..

[23] Kimani-Murage E., Schofield L., Wekesah F., Mohamed S., Mberu B., Ettarh R., Egondi T., Kyobutungi C., Ezeh A. (2014). Vulnerability to Food Insecurity in Urban Slums: Experiences from Nairobi, Kenya. J. Urban Health.

[24] Chandrasekhar S., Aguayo V.M., Krishna V., Nair R. (2017). Household food insecurity and children’s dietary diversity and nutrition in India. Evidence from the comprehensive nutrition survey in Maharashtra. Matern. Child Nutr..

[25] Central Statistical Agency (CSA) Ethiopia, ICF (2016). Ethiopia Demographic and Health Survey 2016.

[26] Amare Z.Y., Ahmed M.E., Mehari A.B. (2019). Determinants of nutritional status among children under age 5 in Ethiopia: Further analysis of the 2016 Ethiopia demographic and health survey. Glob. Health.

[27] Mohammed S.H., Muhammad F., Pakzad R., Alizadeh S. (2019). Socioeconomic inequality in stunting among under-5 children in Ethiopia: A decomposition analysis. BMC Res. Notes..

[28] Spaliviero M., Cheru F. (2017). State of Addis Ababa 2017: The Addis Ababa We Want. UN- Habitat. https://unhabitat.org/books/the-state-of-addis-ababa-2017-the-addis-ababa-we-want/.

[29] The World Bank Ethiopia at a Glance-Overview. https://www.worldbank.org/en/country/ethiopia/overview.

[30] Central Statistical Agency (CSA) Ethiopia (2013). Population Projections of Ethiopia 2007–2037.

[31] Central Statistics Agency (2008). Summary and Statistical Report of the 2007 Population and Housing Census: Population Size by Age and Sex.

[32] The World Bank Enhancing Urban Resilience. https://www.worldbank.org/en/topic/urbandevelopment/publication/addis-ababa-ethiopia-enhancing-urban-resilience.

[33] World Health Organization PROCESS of Translation and Adaptation of Instruments. https://www.who.int/substance_abuse/research_tools/translation/en/.

[34] Cogill B. (2001). Anthropometric Indicators Measurement Guide.

[35] StataCorp (2015). Stata Statistical Software: Release 14.

[36] World Health Organization (2008). Training Course on Child Growth Assessment.

[37] World Education News Reviews Education in Ethiopia. https://wenr.wes.org/2018/11/education-in-ethiopia.

[38] Rutstein S.O. (2015). Steps to Constructing the New DHS Wealth Index.

[39] Coates J., Swindale A., Bilinsky P. (2007). Household Food Insecurity Access Scale (HFIAS) for Measurement of Household Food Access: Indicator Guide (v. 3).

[40] Kennedy G., Ballard T., Dop M.-C. (2011). Guidelines for Measuring Household and Individual Dietary Diversity.

[41] World Health Organization, UNICEF, USAID, AED, UCDAVIS, IFPRI (2010). Indicators for assessing infant and young child feeding practices: Part 2: Measurement. https://www.who.int/nutrition/publications/infantfeeding/9789241599290/en/.

[42] World Health Organization (2011). WHO Anthro for Personal Computers. https://www.who.int/childgrowth/software/en/.

[43] United Nations (2019). The Sustainable Development Goals Report 2019.

[44] De Onis M., Borghi E., Arimond M., Webb P., Croft T., Saha K., De-Regil L.M., Thuita F., Heidkamp R., Krasevec J. (2018). Prevalence thresholds for wasting, overweight and stunting in children under 5 years. Public Health Nutr..

[45] Gebrie A., Alebel A., Zegeye A., Tesfaye B., Ferede A. (2018). Prevalence and associated factors of overweight/ obesity among children and adolescents in Ethiopia: A systematic review and meta-analysis. BMC Obes..

[46] Ethiopian Public Health Institute (2013). Ethiopia National Food Consumption Survey.

[47] Mekonnen N., Asfaw S., Mamo A., Mulu Y., Fentahun N. (2018). Barriers and facilitators of child-feeding practice in a small sample of individuals from Gozamin District, Northwest of Ethiopia: A qualitative study. BMC Nutr..

[48] Solomon D., Aderaw Z., Tegegne T.K. (2017). Minimum dietary diversity and associated factors among children aged 6–23 months in Addis Ababa, Ethiopia. Int. J. Equity Health.

[49] Khattak U.K., Iqbal S.P., Ghazanfar H. (2017). The Role of Parents’ Literacy in Malnutrition of Children Under the Age of Five Years in a Semi-Urban Community of Pakistan: A Case-Control Study. Cureus.

[50] Kandala N.-B., Madungu T.P., Emina J.B., Nzita K.P., Cappuccio F.P. (2011). Malnutrition among children under the age of five in the Democratic Republic of Congo (DRC): Does geographic location matter?. BMC Public Health.

[51] Abuya B.A., Ciera J., Kimani-Murage E. (2012). Effect of mother’s education on child’s nutritional status in the slums of Nairobi. BMC Pediatr..

[52] Corsi D.J., Mejía-Guevara I., Subramanian S.V. (2016). Risk factors for chronic undernutrition among children in India: Estimating relative importance, population attributable risk and fractions. Soc. Sci. Med..

[53] Smith Greenaway E., Leon J., Baker D.P. (2012). Understanding the association between maternal education and use of health services in Ghana: Exploring the role of health knowledge. J. Biosoc. Sci..

[54] Ickes S.B., Hurst T.E., Flax V.L. (2015). Maternal Literacy, Facility Birth, and Education Are Positively Associated with Better Infant and Young Child Feeding Practices and Nutritional Status among Ugandan Children. J. Nutr..

[55] Shroff M., Griffiths P., Adair L., Suchindran C., Bentley M. (2009). Maternal autonomy is inversely related to child stunting in Andhra Pradesh, India. Matern. Child Nutr..

[56] Cunningham K., Ruel M., Ferguson E., Uauy R. (2015). Women’s empowerment and child nutritional status in South Asia: A synthesis of the literature: Women’s empowerment and child nutrition: South Asia. Matern. Child Nutr..

[57] Mutisya M., Kandala N., Ngware M.W., Kabiru C.W. (2015). Household food (in)security and nutritional status of urban poor children aged 6 to 23 months in Kenya. BMC Public Health.

[58] Lo Y.-T., Chang Y.-H., Lee M.-S., Wahlqvist M.L. (2009). Health and nutrition economics: Diet costs are associated with diet quality. Asia Pac. J. Clin. Nutr..

[59] Hawkes C. (2006). Uneven dietary development: Linking the policies and processes of globalization with the nutrition transition, obesity and diet-related chronic diseases. Glob. Health..

[60] Ford N.D., Patel S.A., Narayan K.M.V. (2017). Obesity in Low- and Middle-Income Countries: Burden, Drivers, and Emerging Challenges. Annu. Rev. Public Health..

[61] Reilly J.J. (2008). Physical activity, sedentary behaviour and energy balance in the preschool child: Opportunities for early obesity prevention: Symposium on ‘Behavioural nutrition and energy balance in the young’. Proc. Nutr. Soc..

[62] Wamani H., Åstrøm A.N., Peterson S., Tumwine J.K., Tylleskär T. (2007). Boys are more stunted than girls in Sub-Saharan Africa: A meta-analysis of 16 demographic and health surveys. BMC Pediatr..

[63] Muenchhoff M., Goulder P.J.R. (2014). Sex Differences in Pediatric Infectious Diseases. J. Infect Dis..

[64] Tumilowicz A., Habicht J.-P., Pelto G., Pelletier D.L. (2015). Gender perceptions predict sex differences in growth patterns of indigenous Guatemalan infants and young children. Am. J. Clin. Nutr..

[65] World Health Organization (2017). Double-Duty Actions for Nutrition: Policy Brief.

[66] Makoka D., Masibo P.K. (2015). Is there a threshold level of maternal education sufficient to reduce child undernutrition? Evidence from Malawi, Tanzania and Zimbabwe. BMC Pediatr..

[67] Menon S., Peñalvo J.L. (2019). Actions Targeting the Double Burden of Malnutrition: A Scoping Review. Nutrients.

[68] Terfa B.K., Chen N., Liu D., Zhang X., Niyogi D. (2019). Urban Expansion in Ethiopia from 1987 to 2017: Characteristics, Spatial Patterns, and Driving Forces. Sustainability.

